# Systematic Breakfast Consumption of Medium-Quantity and High-Quality Food Choices Is Associated with Better Vascular Health in Individuals with Cardiovascular Disease Risk Factors

**DOI:** 10.3390/nu15041025

**Published:** 2023-02-18

**Authors:** Eirini D. Basdeki, Antonios A. Argyris, Olga Efthymiou, Elpida Athanasopoulou, Petros P. Sfikakis, Athanase D. Protogerou, Kalliopi Karatzi

**Affiliations:** 1 Cardiovascular Prevention & Research Unit, Clinic & Laboratory of Pathophysiology, Department of Medicine, National and Kapodistrian University of Athens, 11527 Athens, Greece; 2 Department of Life Sciences, School of Sciences, European University of Cyprus, Nicosia 22006, Cyprus; 3 1st Department of Propaedeutic Internal Medicine, Medical School, National and Kapodistrian University of Athens, 11527 Athens, Greece; 4 Laboratory of Dietetics and Quality of Life, Department of Food Science & Human Nutrition, Agricultural University of Athens, 75 Iera Odos, 11855 Athens, Greece

**Keywords:** subclinical vascular damage, breakfast consumption, breakfast quality, blood pressure, cardiovascular disease, Aix, PWV

## Abstract

Background: Breakfast consumption has been associated with the improvement of many cardiovascular disease (CVD) risk factors, yet data regarding its association with subclinical vascular damage, which precedes the onset of CVD, are scarce. The aim of this study is to investigate this association in a large sample of adults with CVD risk factors. Methods: Anthropometric measurements, vascular biomarkers and dietary intake with two 24-h dietary recalls, focusing on breakfast frequency and its quantity and content, were assessed in 902 adults (45.2% males). Breakfast quality was assessed by identifying a posteriori breakfast dietary pattern (DP) by using principal component analysis (PCA). Results: Systematic breakfast consumption (SBC) was inversely associated with central systolic blood pressure (b: −3.28, 95% C.I.: −5.7 to −0.86), diastolic blood pressure (b: −1.85, 95% C.I.: −3.34 to −0.36), augmentation index (b: −3.17, 95% C.I.:−4.98 to 1.35) and left carotid intima media thickness (b: −0.03, 95% C.I.:−0.06 to −0.01) compared to breakfast skipping independently of age, sex, hypertension, diabetes, dyslipidemia, smoking, and BMI. SBC of 10–20% of daily total energy intake (dTEI) was inversely associated with Aix (b: −2.31, 95% C.I.:−4.05 to −0.57) compared to <10% dTEI after adjustment for the aforementioned confounders. DP1 (high coffee and sugar consumption, low consumption of low- and full-fat dairy products, fruits, and fresh juices) was positively associated with Aix (b: 1.19, 95% C.I.: 0.48 to 1.90). Conclusion: SBC comprised of medium-energy density and high-nutrient content food items may be a simple daily habit associated with better vascular health.

## 1. Introduction

Breakfast is defined either as the first meal of the day which breaks the night fast, or the meal that is consumed within 2–3 h after waking, including at least one food item or drink and can be consumed in any location [1]. Moreover, its caloric content usually does not exceed 20–35% of daily total energy intake (dTEI) [2]. Breakfast consumption has been associated with a variety of beneficial health effects, such as lower body mass index (BMI) and better insulin sensitivity, which are significant risk factors for cardiovascular disease (CVD) and Type 2 diabetes mellitus [2]. Moreover, breakfast skipping has been associated with increased risk for CVD or development of risk factors for CVD [3,4,5]. Another important aspect of breakfast consumption is its content in high-quality food items, which is positively associated with mental health, cardiometabolic indices and quality of life, and inversely associated with anxiety and depression [6,7,8].

Early detection of preclinical vascular abnormalities is an important step for efficient prevention of CVD, a leading cause of global mortality [9,10]. Several hemodynamic and vascular biomarkers exist that depict arterial functional and structural damage. Of these, the most commonly used are peripheral and central blood pressure (BP) (systolic and diastolic (SBP, DBP)), augmentation index (Aix) (pressure wave reflections), and pulse wave velocity (PWV) (arterial stiffness), as indices of vascular function, and carotid intima media thickness (IMT) as an index of arterial remodeling and structural damage [10,11,12].

Until now, several markers of subclinical vascular damage (SVD) have been associated with unhealthy dietary behavior; however the possible association between breakfast consumption and subclinical vascular biomarkers has been understudied [13,14]. The only two available studies included a mixed adult population and assessed only breakfast consumption, but not the quality or quantity of the breakfast, and limited vascular biomarkers were employed. Both of them concluded that skipping breakfast was inversely associated with arterial stiffness and atheromatosis.

The aim of the present study was to investigate the potential association between breakfast consumption and vascular health in a large sample of adults with at least one CVD risk factor. Our hypothesis was that breakfast consumption is associated with better vascular structure and function. To test this hypothesis, the frequency, the quantity, and the quality of breakfast were taken into consideration, and multiple vascular indices were employed regarding arterial function and structure.

## 2. Materials and Methods

### 2.1. The Study

The present cross-sectional study was conducted at the Cardiovascular Prevention and Research Unit, Athens, Greece, in accordance with the Declaration of Helsinki and its later amendments and was approved by the Bioethics Committee, and all participants provided written informed consent before entering the study.

### 2.2. Study Population

In the present study, 902 adults participated (45.2% males), with one or more CVD risk factor, but free of established CVD. Exclusion criteria were cancer, liver disease, or eating disorders. All patients abstained from food, drink, and any medication 12 h prior to their visit for anthropometric and vascular measurements.

### 2.3. Definition of CVD Risk Factors

Hypertension was defined by the use of antihypertensive drugs and/or office BP measurement higher than 139/89 mm Hg. Dyslipidemia was defined on the basis of treatment with lipid-modifying drugs or low-density lipoprotein cholesterol level >160 mg/dL [15]. Current smoking was defined by the use of at least one cigarette per day each day of the week; ex-smoking was defined as interruption for more than six months. Family history of premature CVD was defined as the presence of coronary heart disease in a first-degree relative under the age of 55 years for males and 65 years for females [16].

### 2.4. Anthropometric Measurements

A trained dietician performed a physical examination on every participant of the study, using the same protocol and equipment. Participants’ body weight was measured without shoes or heavy clothes to the nearest 10 g by using the Tanita Body Composition Analyzer, BC-418 weight scale. Participants’ height was measured without shoes, with the participants standing with their shoulders relaxed, their arms hanging freely, and their head in Frankfurt horizontal plane, to the nearest 0.1 cm by using stadiometer SECA 213. Body mass index (BMI) was calculated by using both measurements as weight/(height2) (Kg/m^2^).

### 2.5. Vascular Assessment

All participants underwent vascular assessment, performed by the same examiner. BP was assessed, after a 10-min rest in supine position and the average of three sequential readings with a one-min interval was used in the analysis (MicrolifeWatchBP Office, Microlife AG, Widnau, Switzerland). Central BP, Aix (also normalized for the heart rate of 75 bpm -Aix) and carotid to femoral PWV were assessed by a common tonometric method and the use of a generalized transfer function (Sphygmocor; Atcor Medical, Sydney, Australia), as previously described [17]. High-resolution ultrasonography was performed at the carotid and femoral arteries for the assessment of IMT (Vivid 7 Pro, General Electric Healthcare, Little Chalfont, United Kingdom). IMT was calculated in the right and left common carotid artery and the carotid bulb and internal carotid artery, as well as at the femoral artery. Two sequential images were obtained, and the average measurements were used [18].

### 2.6. Dietary Intake Assessment and Definition of Breakfast Consumption

Two 24-h dietary recalls were conducted (one weekday and one weekend day) with an interval of at least one week in between, by trained dieticians. Data were analyzed in food groups, where separation was based on food equivalents with further separation in subgroups where needed. Moreover, analysis of energy content, macronutrients, and micronutrients was conducted by using Nutritionist Pro software (Axxya Systems Nutritionist Pro TM 2011). Breakfast consumption was defined as the first meal of the day consumed in the morning after a night’s sleep. Systematic breakfast skipping was defined as consumption of breakfast ≤1 times out of 2 total 24 h recalls, and breakfast consumption in both recalls was considered as systematic breakfast consumption. Additionally, consumption of solely coffee was not considered as breakfast consumption. Breakfast quality was indirectly assessed by identifying a posteriori dietary patterns.

### 2.7. Statistical Analysis

Statistical analysis was conducted by using statistical package 21.0 (SPSS: Statistical Package for Social Sciences, SPSS Inc., Chicago, IL, USA). The level of statistical significance was set at *p* ≤ 0.05 for all analyses. Normality of distribution of continuous variables was checked by using the Kolmogorov–Smirnov test. Categorical variables are presented as relative frequencies (%) and continuous as mean ± standard deviation (SD). 

Breakfast caloric intake was divided into three categories depending on the percentage of dTEI consumed in that meal: systematic breakfast consumption of >0–10% of dTEI (first category); systematic breakfast consumption of 10–20% of dTEI (second category); systematic breakfast consumption of >20% of dTEI (third category) [19].

Differences between systematic breakfast consumers and systematic breakfast skippers were tested with independent sample *t*-tests for continuous variables and with chi-square for categorical variables. Analysis of variance (one-way ANOVA) was conducted to address differences between the three breakfast categories (related to the % of dTEI consumed on breakfast) among systematic breakfast consumers. 

Multiple linear regression analysis was performed to determine the independent associations between vascular biomarkers and systematic breakfast consumption. Results are presented as standardized beta coefficients and 95% confidence intervals (CI) (for peripheral and central SBP, DBP, PWV, Aix, right and left IMT). Moreover, univariate analysis of variance was performed to check the associations between vascular biomarkers and categories of breakfast according to percentages of dTEI consumed at breakfast. Three different models were applied to assess all of the above associations:

model 1: adjusted for age and sex;
model 2: adjusted for age, sex, hypertension, diabetes mellitus, dyslipidemia, and smoking; and
model 3: adjusted for age, sex, hypertension, diabetes mellitus, dyslipidemia, smoking, and BMI.

Principal component analysis (PCA) was applied to identify dietary patterns (DP) among study participants with systematic breakfast consumption. The Kaiser–Meyer–Olkin (KMO) criterion was applied, and it was equal to 0.432. The orthogonal rotation (varimax option) was used to derive optimal noncorrelated components (DPs) and the Bartlett’s method was used to estimate factor scores. The selection of the optimal number of components was based on an eigenvalue >1 (Kaiser criterion), and it was further corroborated by visual assessment of the scree plot, retaining only components on the steep slope. If one of the initial variables correlated with more than one component, it participated in the interpretation of the DP in which it displayed the highest coefficient value. Three DPs emerged after PCA. Multiple linear regression analysis was applied to determine possible associations between DPs and subclinical vascular biomarkers. Results are presented as standardized beta coefficients and 95% confidence intervals (CI) (for peripheral and central SBP, DBP, PWV, Aix, and right and left IMT). The aforementioned models that were applied in breakfast quantity analyses were also applied, in order to assess the above associations regarding breakfast quality.

## 3. Results

The present study included 902 subjects (45.2% males) with at least one risk factor for CVD, mean age 52.4 ± 13.8 years, and mean BMI 27.97 ± 5.52 Kg/m^2^. The total sample consisted of 765 systematic and 136 nonsystematic breakfast consumers. Nonsystematic breakfast consumers had significantly higher total cholesterol (*p* = 0.003), LDL (*p* = 0.002), triglycerides (*p* = 0.01), and DBP levels (*p* = 0.026) compared to systematic breakfast consumers. On the other hand, systematic breakfast consumers had significantly higher daily energy intake levels (*p* = 0.000). Table 1 summarizes characteristics of the total study sample and systematic and nonsystematic breakfast consumers. 

Table 2 summarizes vascular biomarkers of systematic breakfast consumers (*n* = 765) according to percentage of energy intake consumed at breakfast. There were no differences in vascular indices between the three groups, except for PWV. Specifically, PWV tended to be higher (*p* = 0.051) in the group that consumed >20% compared to the group that consumed >0–10% of dTEI at breakfast. 

Data regarding dietary intake of systematic breakfast consumers according to dTEI at breakfast is shown in Table 3. Participants at the lowest group of dTEI at breakfast (>0–10% of dTEI) consumed a significantly higher percentage of energy from fat (*p* = 0.029) and had higher dTEI (*p* < 0.001). Few differences between the three groups were noted regarding food groups. Specifically, breakfast consumers with >20% of dTEI at breakfast consumed significantly more low-fat (*p* = 0.004) and full-fat (*p* = 0.006) dairy products and more fruits (*p* = 0.019) compared to the group that consumed >0–10% of dTEI at breakfast.

Associations of subclinical vascular biomarkers and systematic breakfast consumption are presented in Table 4. Systematic breakfast consumption was inversely associated with central SBP (B: −3.28, 95% C.I.: −5.7–(−0.86)), DBP (B: −1.85, 95% C.I.: −3.34–(−0.36)), Aix (B: −3.17, 95% C.I.: −4.98–1.35), and left IMT (B: −0.03, 95% C.I.: −0.06–(−0.01)) independently of age, sex, hypertension, diabetes, dyslipidemia, smoking, and BMI. 

Table 5 shows the associations between vascular biomarkers and systematic breakfast consumption categories according to percentage of dTEI consumed at breakfast. Consumption of 10–20% of dTEI at breakfast was inversely associated with Aix levels (B: −2.31, 95% C.I.: −4.05–(−0.57)) after adjustment for all possible confounders, as presented above.

Table 6 presents the three DPs that emerged from PCA analysis. DP1 consists of high coffee and sugar consumption, low consumption of low-fat and full-fat dairy products and low consumption of fruits and fresh juices; DP2 consists of high consumption of refined grains, cold cuts and full-fat cheese and DP3 consists of high consumption of sugar/honey/jam substitutes, whole wheat grains and tahini/peanut butter and margarine.

Associations between the three identified DPs and subclinical vascular biomarkers are presented in Table 7. DP1 was positively associated with Aix (B: 1.19, 95% C.I.: 0.48–1.90) independently of age, sex, hypertension, diabetes, dyslipidemia, smoking, and BMI. It was also positively associated with DBP levels (0.66 (0.10, 1.23)) after adjusting for age, sex, hypertension, diabetes, dyslipidemia, and smoking, but after adding BMI to the model significance was lost. DP2 and DP3 showed no statistically significant associations with subclinical vascular biomarkers.

## 4. Discussion

The present study aimed to examine the possible association between breakfast consumption and SVD in adults, free of overt CVD but with at least one CVD risk factor. Both breakfast quality and quantity were examined: quantity as percentage of dTEI consumed at breakfast and quality through a posteriori breakfast dietary patterns. The main findings of this study are: (i) the positive association of systematic breakfast consumption with better hemodynamic indices compared to breakfast skipping; (ii) the inverse association of moderate compared to lower energy consumption at breakfast with Aix; and (iii) the positive association of DP1 (high coffee and sugar consumption, low consumption of low-fat and full-fat dairy products, low consumption of fruits and fresh juices) with Aix.

Systematic breakfast consumption was found to be inversely associated with central SBP, peripheral DBP, Aix, and IMT compared to breakfast skipping. Previous studies, examining associations between breakfast eating/skipping and BP levels or risk of hypertension, are in agreement with the present findings [20,21]. Interestingly, there are no available studies examining the association of breakfast consumption with arterial stiffness using Aix, but there is a relevant cross-sectional study conducted in adults with established CVD or with CVD risk factors, showing an inverse association between breakfast consumption and PWV, which is in alignment with the findings of the present study [13]. The same study also showed that breakfast skippers had significantly higher carotid IMT compared to systematic breakfast consumers, which is also in line with the present findings. Considering all the mentioned findings, it is possible that systematic breakfast consumption might lead to a reduction not only in peripheral resistance, but also, and mainly, in pressure wave reflection coefficients, resulting in lower pressure wave reflections as quantified by Aix, which in turn decreases aortic pressures. This observation explains why in the present study aortic, but not peripheral SBP, was associated with systematic breakfast consumption. As recently shown in a systematic metanalysis, lower Aix are associated with lower ventricular mass [22], and this may explain the well-described association with reduced mortality [11]. The exact mechanism behind the benefits of systematic breakfast eating on central hemodynamics must be elucidated by future relevant studies.

Among systematic breakfast consumers, it was found that moderate (10–20%) energy intake at breakfast was associated with lower pressure wave reflections (Aix) compared to lower energy intake consumption. There are no available studies examining the impact of breakfast energy content on central haemodynamics, pressure wave reflections, and arterial stiffness. However, there is one cross-sectional study conducted in either healthy middle-aged adults or middle-aged adults having CVD risk factors, which investigated the association of energy consumed at breakfast with atheromatosis [14]. In contrast with the present study, it was found that moderate energy consumption (5–20%) at breakfast had higher risk of subclinical atheromatosis, compared to the group that consumed a higher amount of energy at breakfast. This discrepancy might be attributed to differences in the studied populations (in the aforementioned study, healthy adults were also included), and also to the quite distinct pathologies that induce atheromatosis and modify pressure wave reflections.

Regarding breakfast quality, out of the three DPs that were identified, DP1 (high coffee and sugar consumption, low consumption of low-fat and full-fat dairy products, low consumption of fruits and fresh juices) was positively associated with Aix. There are no available studies investigating the association between DPs at breakfast and vascular health, but some of the DPs’ components have been previously associated with vascular biomarkers. Data regarding coffee consumption and Aix are insufficient, because there are studies in agreement with our findings [23] and studies that found an inverse association between coffee consumption and Aix [24,25]. The positive association of coffee consumption with Aix could be explained by the increased wave reflections caused by caffeine intake, which in turn increase Aix [23]. Dairy product consumption has also been inversely associated with arterial stiffness, which is attributed to bioactive peptides released during milk-protein digestion [26], but there are also opposite findings reported [27]; hence, this issue needs further research. The positive association of low consumption of fruits and fresh juices in the DP with Aix could be supported by the fact that increased concentration of flavonoids in fruits, have been described as having a protective role against unfavorable vascular alterations [28,29].

The present study is the first to assess the association between breakfast quality and SVD. It is also the first study that assessed the association of breakfast consumption by using a large number of subclinical vascular biomarkers. Vascular assessment was conducted by the same examiner with the same equipment, which reduces observer’s variability. Moreover, 24-h recalls were used for the nutritional assessment, which provide useful information regarding participants’ meal patterns, quality, and quantity of food. However, this study has some certain limitations. Its cross-sectional design does not allow us to establish a causative relationship. Furthermore, 24-h recalls are based on participants’ memory and are prone to recall bias. Social desirability bias should also be added to the study’s limitations. Finally, dietary intake analysis was conducted by the use of Nutritionist Pro, a program based on US food databases, and there may be differences in the content of some nutrients compared with the food items consumed in Greece.

## 5. Conclusions

As a conclusion, systematic consumption of a high-quality, moderate-in-energy breakfast is beneficially associated with BP levels and particularly with aortic SBP due to lower pressure wave reflections. Breakfast consumption might be a simple, everyday dietary habit that could promote vascular health; however, these data need to be further investigated and confirmed.

## Figures and Tables

**Table 1:** Descriptive and clinical characteristics of the study population.

	Total Sample (*n* = 902)	Systematic Breakfast Skippers (*n* = 136)	Systematic Breakfast Consumers (*n* = 765)	*p*-Value
Age (years)	52.4 ± 13.8	49.3 ± 12.65	52.95 ± 13.94	**0.004**
Sex (%)				
Male	45.2	53.7	43.7	**0.031**
BMI (Kg/m^2^)	27.97 ± 5.52	28.0 ± 6.32	27.96 ± 5.38	0.947
Smoking (%)				
Never smokers	35.7%	25.7%	39.7%	**<0.001**
Former smokers	39.7%	53.7%	35.6%	
Current smokers	24.6%	20.6%	24.6%	
Biochemical biomarkers				
Fasting blood glucose (mg/dL)	98.73 ± 29.98	102.17 ± 40.76	98.18 ± 27.74	0.205
Total cholesterol (mg/dL)	195.68 ± 36.85	205.54 ± 39.45	194.01 ± 36.18	**0.** **003**
LDL-C (mg/dL)	118.17 ± 31.47	126.98 ± 33.33	116.72 ± 30.93	**0.** **002**
HDL-C (mg/dL)	56.29 ± 17.12	54.68 ± 17.63	56.56 ± 17.04	0.303
TG (mg/dL)	108.43 ± 64.37	123.40 ± 66.29	105.90 ± 63.80	**0.** **010**
Diabetes mellitus (%)				
Type 1 diabetes mellitus	7.9	5.1	8.4	0.132
Type 2 diabetes mellitus	11.6	8.1	12.3	
Hypertension (%)				
Yes	51.2	49.1	51.5	0.638
Dyslipidemia (%)				
Yes	36.9	36.8	36.9	0.983
Subclinical vascular biomarkers				
Peripheral SBP (mmHg)	125.25 ± 15.83	124.90 ± 17.14	125.32 ± 15.61	0.775
Central SBP (mmHg)	114.77 ± 16.37	115.71 ± 16.09	114.62 ± 16.43	0.473
DBP (mmHg)	75.97 ± 9.17	77.59 ± 9.95	75.68 ± 9.01	**0.** **026**
PWV (m/sec)	8.30 ± 1.96	7.98 ± 1.83	8.35 ± 1.98	0.053
Aix (%)	25.97 ± 12.90	26.81 ± 11.07	25.82 ± 13.21	0.410
Right IMT (mm)	0.68 ± 0.15	0.67 ± 0.15	0.68 ± 0.15	0.227
Left IMT (mm)	0.72 ± 0.17	0.73 ± 0.16	0.72 ± 0.17	0.598
Energy intake				
Mean breakfast energy intake (Kcal)	239.67 ± 168.82	84.17 ± 96.46	267.63 ± 163.72	**<0.** **001**
Total energy intake (Kcal)	1709.34 ± 637.17	1579.94 ± 553.73	1732.34 ± 648.51	**0.** **010**
Energy from CHO at breakfast (%)	42.22 ± 9.73	39.60 ± 11.64	42.69 ± 9.28	**0.** **001**
Energy from PRO at breakfast (%)	17.14 ± 4.97	17.19 ± 5.16	17.13 ± 4.94	0.901
Energy from FAT at breakfast (%)	39.85 ± 9.12	40.87 ± 11.05	39.67 ± 8.73	0.155

dTEI, total energy intake; BMI, body mass index; LDL-C, low-density lipoprotein cholesterol; HDL-C, high-density lipoprotein cholesterol; TG, triglycerides; SBP, systolic blood pressure; DBP, diastolic blood pressure; PWV, pulse wave velocity; Aix, augmentation index; IMT, intima media thickness; CHO, carbohydrates; PRO, protein. Bold indicates statistical significance.

**Table 2:** Subclinical vascular biomarkers according to % dTEI consumed at breakfast among systematic breakfast consumers.

	>0–10% of dTEI in Breakfast	10–20% of dTEI in Breakfast	>20% of dTEI in Breakfast	*p*-Value
Peripheral SBP (mmHg)	124.64 ± 15.08	125.04 ± 15.16	126.47 ± 16.86	0.450
Central SBP (mmHg)	113.88 ± 14.28	114.35 ± 16.59	115.82 ± 18.05	0.456
DBP (mmHg)	76.01 ± 9.08	75.46 ± 8.97	75.76 ± 9.04	0.783
PWV (m/sec)	8.22 ± 2.03	8.25 ± 1.79	8.65 ± 2.21	**0.051**
Aix (%)	26.31 ± 13.28	24.80 ± 13.61	27.18 ± 12.31	0.101
Right IMT (mm)	0.69 ± 0.15	0.68 ± 0.15	0.70 ± 0.16	0.285
Left IMT (mm)	0.72 ± 0.15	0.71 ± 0.17	0.73 ± 0.17	0.327

dTEI, total energy intake; SBP, systolic blood pressure; DBP, diastolic blood pressure; PWV, pulse wave velocity; Aix, augmentation index; IMT, intima media thickness. Bold indicates statistical significance.

**Table 3:** Dietary intake according to % dTEI consumed at breakfast among systematic breakfast consumers.

	>0–10% of dTEI in Breakfast	10–20% of dTEI in Breakfast	>20% of dTEI in Breakfast	*p*-Value
Breakfast energy consumption (Kcal)	113.84 ± 73.58	258.63 ± 97.66	432.18 ± 168.20	**<0.001**
Energy from CHO at breakfast (%)	40.25 ± 9.65	42.96 ± 8.29	44.54 ± 10.11	**<0.001**
Energy from PRO at breakfast (%)	16.94 ± 5.87	17.29 ± 4.70	17.04 ± 4.35	0.698
Energy from FAT at breakfast (%)	41.02 ± 8.90	39.42 ± 8.03	38.79 ± 9.60	**0.029**
dTEI (Kcal)	1845.99 ± 777.05	1747.51 ± 624.06	1595.53 ± 521.22	**<0.001**
Food groups				
Low-fat dairy products (250 mL milk or soy milk, 1 cup of yogurt)	0.30 ± 0.47	0.46 ± 0.59	0.47 ± 0.67	**0.004**
Full-fat dairy products (250 mL milk, 1 cup of yogurt)	0.14 ± 0.30	0.19 ± 0.37	0.27 ± 0.53	**0.006**
Vegetables (e.g., 1 medium tomato or pepper, ½ cup of cabbage or peas or lettuce or spinach)	2.49 ± 2.05	2.51 ± 1.89	2.22 ± 1.80	0.193
Legumes cooked and drained (½ cup)	0.27 ± 0.54	0.30 ± 0.68	0.29 ± 0.66	0.857
Fruits (e.g., 1 medium apple or peach or orange or pear, 1 cup of strawberries or melon) and fresh juice (½ cup)	1.31 ± 1.36	1.64 ± 1.62	1.72 ± 1.66	**0.019**
Processed juices (½ cup)	0.06 ± 0.28	0.08 ± 0.32	0.09 ± 0.35	0.714
Refined grains (e.g., 30 g of white bread, ½ cup rice or pasta, 2 rusks)	3.13 ± 2.69	3.05 ± 2.25	2.72 ± 2.27	0.178
Whole grains (e.g., 30 g of whole wheat bread, ½ cup brown or wild rice or whole wheat pasta, 2 rusks)	1.07 ± 1.42	1.23 ± 1.43	1.06 ± 1.46	0.250
Danishes (1 medium slice)	0.13 ± 0.30	0.10 ± 0.27	0.12 ± 0.29	0.470
Low-fat cheese (30 g)	0.17 ± 0.41	0.27 ± 0.53	0.20 ± 0.49	0.057
Full-fat cheese (30 g or 1 slice)	0.95 ± 1.27	0.95 ± 1.09	0.90 ± 1.04	0.847
Dressings, full fat or light (1 tbs)	0.16 ± 0.47	0.13 ± 0.38	0.12 ± 0.41	0.608
Sweets rich in sugar and saturated fat (e.g., 1 slice of cake, 1 ice cream)	0.77 ± 1.04	0.66 ± 0.90	0.75 ± 1.06	0.356
Sugar (1 tsp)	0.82 ± 1.34	0.72 ± 1.09	0.65 ± 1.02	0.287
Sugar substitutes, honey, jam (1 tsp)	0.50 ± 1.07	0.68 ± 1.16	0.58 ± 0.92	0.134

dTEI, total energy intake; CHO, carbohydrates; PRO, proteins; tbs, tablespoon; tsp, teaspoon. Bold indicates statistical significance.

**Table 4:** Associations between subclinical vascular biomarkers and systematic breakfast consumption.

	Model 1		Model 2		Model 3	
	Β (95% CI)	*p*-Value	Β (95% CI)	*p*-Value	Β (95% CI)	*p*-Value
Peripheral SBP	−0.70 (−3.40, 2.00)	0.610	−0.75 (−3.05, 1.54)	0.521	−1.02 (−3.34, 1.29)	0.386
Central SBP	**−3.06** **(−5.73, −0.39)**	**0.025**	**−3.05** **(−5.45, −0.65)**	**0.013**	**−3.28** **(−5.70, −0.86)**	**0.008**
DBP	**−1.82** **(−3.43, −0.21)**	**0.027**	**−1.76** **(−3.24, −0.29)**	**0.019**	**−1.85 ** **(−3.34, −0.36)**	**0.015**
Aix	**−3.65 ** **(−5.47, −1.83)**	**0.000**	**−3.42 ** **(−5.22, −1.62)**	**0.000**	**−3.17 ** **(−4.98, −1.35)**	**0.001**
PWV	0.11 (−1.84, 0.40)	0.462	0.05 (−0.23, 0.32)	0.743	0.05 (−0.23, 0.32)	0.726
Right IMT	−0.01 (−0.03, 0.02)	0.673	−0.00 (−0.03, 0.02)	0.712	−0.00 (−0.03, 0.02)	0.754
Left IMT	**−0.03 ** **(−0.06, −0.01)**	**0.011**	**−0.30 ** **(−0.05, −0.01)**	**0.012**	**−0.03 ** **(−0.06, −0.01)**	**0.007**

SBP, systolic blood pressure; DBP, diastolic blood pressure; PWV, pulse wave velocity; Aix, augmentation index; MT, intima media thickness; BMI, body mass index; Model 1, adjusted for age and sex; Model 2, adjusted for age, sex, hypertension, diabetes mellitus, dyslipidemia, and smoking; Model 3, adjusted for age, sex, hypertension, diabetes mellitus, dyslipidemia, smoking, and BMI. Bold indicates statistical significance.

**Table 5:** Associations between subclinical vascular biomarkers and systematic breakfast consumption categories according to percentage of dTEI consumed at breakfast, using a reference the >0–10% of dTEI subgroup.

	Model 1		Model 2		Model 3	
	Β (95% CI)	*p*-Value	Β (95% CI)	*p*-Value	Β (95% CI)	*p*-Value
Peripheral SBP						
>0–10% of dTEI in breakfast	1.00		1.00		1.00	
10–20% of dTEI in breakfast	0.55 (−1.95, 3.06)	0.664	−0.55 (−2.70, 1.59)	0.615	−0.45 (−2.59, 1.70)	0.683
>20% of dTEI in breakfast	0.63 (−2.20, 3.47)	0.662	0.48 (−1.94, 2.90)	0.697	0.74 (−1.70, 3.17)	0.554
Central SBP						
>0–10% of dTEI in breakfast	1.00		1.00		1.00	
10–20% of dTEI in breakfast	0.50 (−2.01, 3.00)	0.697	−0.47 (−2.74, 1.80)	0.683	−0.39 (−2.66, 1.89)	0.740
>20% of dTEI in breakfast	0.14 (−2.71, 2.99)	0.922	0.09 (−2.47, 2.66)	0.944	0.31 (−2.28, 2.90)	0.815
DBP						
>0–10% of dTEI in breakfast	1.00		1.00		1.00	
10–20% of dTEI in breakfast	−0.38 (−1.88, 1.12)	0.615	−1.00 (−2.39, 0.39)	0.158	−0.96 (−2.36, 0.43)	0.176
>20% of dTEI in breakfast	−0.44 (−2.14, 1.26)	0.611	−0.60 (−2.16, 0.97)	0.455	−0.50 (−2.08, 1.08)	0.535
Aix						
>0–10% of dTEI in breakfast	1.00		1.00		1.00	
10–20% of dTEI in breakfast	**−1.89 ** **(−3.64, −0.14)**	**0.034**	**−2.19 ** **(−3.94, −0.45)**	**0.014**	**−2.31 ** **(−4.05, −0.57)**	**0.010**
>20% of dTEI in breakfast	−0.93 (−2.92, 1.06)	0.358	−0.10 (−2.97, 0.97)	0.320	−1.29 (−3.28, 0.69)	0.201
PWV						
>0–10% of dTEI in breakfast	1.00		1.00		1.00	
10–20% of dTEI in breakfast	0.09 (−0.20, 0.37)	0.554	0.07 (−0.20, 0.33)	0.613	0.07 (−0.20, 0.33)	0.618
>20% of dTEI in breakfast	0.20 (−0.12, 0.52)	0.210	0.24 (−0.06, 0.53)	0.117	0.23 (−0.07, 0.53)	0.125
Right IMT						
>0–10% of dTEI in breakfast	1.00		1.00		1.00	
10–20% of dTEI in breakfast	−0.01 (−0.03, 0.01)	0.334	−0.01 (−0.03, 0.01)	0.237	−0.01 (−0.03, 0.01)	0.221
>20% of dTEI in breakfast	−0.01 (−0.04, 0.01)	0.272	−0.01 (−0.04, 0.01)	0.269	−0.01 (−0.04, 0.01)	0.236
Left IMT						
>0–10% of dTEI in breakfast	1.00		1.00		1.00	
10–20% of dTEI in breakfast	−0.01 (−0.03, 0.02)	0.505	−0.01 (−0.03, 0.02)	0.538	−0.01 (−0.03, 0.02)	0.574
>20% of dTEI in breakfast	−0.01 (−0.04, 0.01)	0.341	−0.01 (−0.04, 0.02)	0.445	−0.01 (−0.03, 0.02)	0.514

dTEI, daily total energy intake; SBP, systolic blood pressure; DBP, diastolic blood pressure; PWV, pulse wave velocity; Aix, augmentation index; IMT, intima media thickness; BMI, body mass index; Model 1, adjusted for age and sex; Model 2, adjusted for age, sex, hypertension, diabetes mellitus, dyslipidemia, and smoking; Model 3, adjusted for age, sex, hypertension, diabetes mellitus, dyslipidemia, smoking, and BMI. Bold indicates statistical significance.

**Table 6:** Identification of breakfast dietary patterns.

Food Groups	Dietary Pattern 1	Dietary Pattern 2	Dietary Pattern 3
Consumption of coffee	0.746	-	-
Consumption of sugar	0.712	-	-
Consumption of low-fat/full-fat dairy products	−0.426	-	-
Consumption of fruits and fresh juices	−0.380	-	-
Consumption of refined grains	-	0.723	-
Consumption of cold cuts	-	0.638	-
Consumption of full-fat cheese	-	0.589	-
Consumption of sugar/honey/jam substitutes	-	-	0.757
Consumption of whole wheat grains	-	-	0.616
Consumption of tahini/peanut butter/margarine	-	-	0.492
Explained variance %	15,023	14,356	13,028

Variables with the highest factor loading (>|0.3|) within the component.

**Table 7:** Multiple linear regression analysis between the three identified breakfast dietary patterns and subclinical vascular biomarkers.

	Model 1		Model 2		Model 3	
	Β (95% CI)	*p*-Value	Β (95% CI)	*p*-Value	Β (95% CI)	*p*-Value
Peripheral SBP						
DP1	−0.40 (−1.42, 0.62)	0.441	0.52 (−1.39, 0.36)	0.249	−0.48 (−1.36, 0.40)	0.285
DP2	**1.04 ** **(0.02, 2.07)**	**0.046**	0.50 (−0.38, 1.37)	0.267	0.47 (−0.41, 1.34)	0.297
DP3	−0.35 (−1.38, 0.67)	0.499	−0.29 (−1.17, 0.59)	0.517	0.40 (−1.29, 0.49)	0.377
Central SBP						
DP1	0.09 (−0.94, 1.11 )	0.870	0.05 (−0.89, 0.97)	0.925	0.08 (−0.85, 1.01)	0.868
DP2	**0.52 ** **(0.11, 2.16)**	**0.030**	0.65 (−0.28, 1.58)	0.171	0.62 (−0.31, 1.35)	0.190
DP3	−0.38 (−1.40, 0.65)	0.471	−0.37 (−1.29, 0.56)	0.439	−0.47 (−1.41, 0.47)	0.331
DBP						
DP1	**0.81 ** **(0.20, 1.42)**	**0.010**	**0.66 ** **(0.10, 1.23)**	**0.022**	0.68 (0.12, 1.25)	0.992
DP2	0.40 (−0.21, 1.02)	0.201	0.18 (−0.39, 0.75)	0.539	0.17 (−0.41, 0.74)	0.570
DP3	−0.22 (−0.83, 0.40)	0.485	−0.27 (−0.83, 0.30)	0.360	−0.32 (−0.89, 0.26)	0.280
Aix						
DP1	**1.35** **(0.64, 2.06)**	**<0.001**	**1.23 ** **(0.52, 1.94)**	**0.001**	**1.19 ** **(0.48, 1.90)**	**0.001**
DP2	0.42 (−0.30, 1.14)	0.252	0.30 (−0.41, 1.02)	0.403	0.34 (−0.37, 1.06)	0.346
DP3	−0.09 (−0.81, 0.63)	0.805	−0.16 (−0.88, 0.56)	0.660	−0.05 (−0.77, 0.68)	0.904
PWV						
DP1	−0.08 (−0.19, 0.04)	0.181	−0.07 (−0.17, 0.04)	0.225	−0.07 (−0.17, 0.04)	0.218
DP2	0.09 (−0.02, 0.21)	0.113	0.06 (−0.04, 0.17)	0.245	0.07 (−0.04, 0.17)	0.235
DP3	−0.06 (−0.17, 0.05)	0.305	−0.06 (−0.16, 0.05)	0.299	−0.06 (−0.16, 0.05)	0.319
Right IMT						
DP1	0.00 (−0.01, 0.01)	0.411	−0.01 (−0.00, 0.01)	0.230	0.01 (−0.00, 0.01)	0.243
DP2	0.01 (−0.00, 0.02)	0.066	0.01 (−0.00, 0.01)	0.228	0.01 (−0.00, 0.01)	0.218
DP3	−0.01 (−0.02, 0.02)	0.122	−0.01 (−0.01, 0.00)	0.171	−0.01 (−0.01, 0.00)	0.194
Left IMT						
DP1	0.00 (−0.01, 0.01)	0.972	0.00 (−0.01, 0.01)	0.771	0.00 (−0.01, 0.01)	0.730
DP2	0.01 (−0.00, 0.02)	0.074	0.01 (−0.00, 0.02)	0.213	0.01 (−0.00, 0.01)	0.229
DP3	−0.00 (−0.01, 0.01)	0.641	−0.00 (−0.01, 0.01)	0.991	−0.00 (−0.01, 0.01)	0.894

DP, dietary pattern; SBP, systolic blood pressure; DBP, diastolic blood pressure; PWV, pulse wave velocity; Aix, augmentation index; IMT, intima media thickness; BMI, body mass index; Model 1, adjusted for age and sex; Model 2, adjusted for age, sex, hypertension, diabetes mellitus, dyslipidemia, and smoking; Model 3, adjusted for age, sex, hypertension, diabetes mellitus, dyslipidemia, smoking, and BMI. Bold indicates statistical significance.

## Data Availability

Data can be provided after request.
